# Patient-reported significant disability after major traumatic upper extremity amputation

**DOI:** 10.1177/17531934231215791

**Published:** 2023-11-22

**Authors:** Joonas Pyörny, Margit Karelson, Ida Neergård Sletten, Anniina Ukkola, Jarkko Jokihaara

**Affiliations:** 1 Faculty of Medicine and Health Technology, Tampere University, Finland; 2 Center for Musculoskeletal Diseases, Tampere University Hospital, Tampere, Finland; 3 Division of Orthopaedic Surgery, Oslo University Hospital, Oslo, Norway

**Keywords:** Amputation, replantation, upper extremity, traumatic

## Abstract

The aims of this study were to record patient-reported outcomes of treatment of proximal upper extremity amputation injuries and subsequent return to work. A consecutive cohort of 38 patients with a traumatic amputation at or proximal to the carpus had been treated with a replantation or revision (completion) amputation in Tampere University Hospital between 2009 and 2019, and 31 of them participated in this study. The primary outcome was the Disabilities of the Arm, Shoulder and Hand Outcome Measure (DASH). Patients reported significant disability indicated by DASH score after replantation (median 30; interquartile range [IQR] 21–47) and revision (completion) amputation (median 33; IQR 16–52). Most patients had cold intolerance and reported low hand function and aesthetics scores. Out of 17 working patients, 10 did not return to their previous work. Our study demonstrates the influence of major upper extremity amputation on daily life activities, even after a successful replantation.

**Level of evidence**: IV

## Introduction

A major upper extremity amputation, defined as an amputation at or proximal to the carpus (Behrend et al., 2011) is classified as total (division of all structures) or subtotal (fracture or exarticulation with loss of circulation, but with some tissue in-continuity). Replantation and revascularization are established treatments to restore vitality and function (Pierrie et al., 2018). When a replantation is not technically feasible or unsuccessful, the limb is treated by a revision (completion) amputation (Larson et al., 2013). A mechanism of severe avulsion or crush injury, multi-level amputation, extensive soft-tissue or nerve damage, or both, and contamination are all factors associated with fewer replantation attempts (Larson et al., 2013; Nawijn et al., 2020).

The survival proportion after replantation and revascularization is over 85% (Gulgonen and Ozer, 2011; Laing et al., 2012) but the functional outcome is uncertain (Lin et al., 2012; Wang et al., 2020). Recovery from extensive multi-tissue damage and long-distance nervous reinnervation is unpredictable and directly related to the level of the injury (Lin et al., 2012; Otto et al., 2015; Ramji et al., 2020; Resnik et al., 2022). Patient-reported outcome measures (PROMs) are the most useful assessments for disability and symptoms in musculoskeletal conditions (Makhni, 2021; Motion Group, 2018; Resnik et al., 2017; Weinfurt and Reeve, 2022). However, most previous studies have reported objective results after amputations (Atzei et al., 2005; Battiston et al., 2007; Cavadas et al., 2009; Gulgonen and Ozer, 2011; Hoang et al., 2009; Laing et al., 2012; Leclère et al., 2012; Ramji et al., 2020; Sabapathy et al., 2007; Wang et al., 2020) and there are only a few studies with validated PROMs (Assouline et al., 2017; Mattiassich et al., 2017; Ng et al., 2014; Pet et al., 2016; Resnik et al., 2019, 2020, 2022; Stanger et al., 2015).

The primary aim of this study was to record upper extremity disability and pain, health-related quality of life, cold intolerance and appearance in patients with major upper extremity amputations. The secondary aim was to describe the patients’ return to previous work.

## Methods

### Study design and setting

In this retrospective descriptive cohort study, we screened all patients who had sustained a traumatic major upper extremity amputation between the years 2009 and 2019. We identified eligible patients using diagnostic and treatment codes from the electronic medical records of Tampere University Hospital, which provides replantation surgery for a referral area with approximately 3 million people. The inclusion criteria were a traumatic injury at or proximal to the carpus, which had caused a fracture or exarticulation, and loss of circulation distal to the injury. During the study period, replantation or revascularization was always done if technically feasible. There were no exclusion criteria.

This study was conducted according to the guidelines of the Institutional Review Board of the Pirkanmaa Hospital District, Finland.

### Participants

All eligible patients were invited to participate in the study. Information about their age, sex, comorbidities, hand dominance, amputation level, injury mechanism, surgical treatment and complications was collected from the hospital’s electronic medical record. The PROMs were sent by mail and non-respondents received two reminders. To describe outcomes between different treatments, we grouped patients into successful replantation/revascularization and revision amputation. The latter group included primary revision (completion) amputation (if replantation was not feasible) and secondary revision amputation after an unsuccessful replantation attempt. Patients were also sorted according to injury level: either distal to the elbow joint or proximal to, or through, the elbow joint.

### Outcomes

The primary outcome was the Disability of Arm, Shoulder and Hand Outcome Measure (DASH), a 30-item generic PROM for upper extremity disability, which is appropriate for patients with traumatic amputations (Giladi et al., 2014). The raw score is converted to a score on a scale of 0–100, in which higher numbers indicate less function and more pain (Hudak et al., 1996). The minimum clinically important difference (MCID) for DASH is estimated to be 10 (95% confidence interval [CI]: 7 to 14) (Gummesson et al., 2003). We used a score of 20 as the cut-off value to identify patients with clinically relevant disability, based on the sum of the MCID and population normal values (Aasheim and Finsen, 2013; Gkotsi et al., 2021; Hunsaker et al., 2002).

The secondary outcomes were:

- The health-related quality of life was measured by the EuroQol EQ-5D-5L index and EuroQol visual analogue scale (EQ-VAS; from 0, the worst imaginable health state, to 100, the best imaginable health state) (Slobogean et al., 2010). Because Finnish population values are unavailable for the EQ-5D-5L, we used the values from the culturally and socioeconomically similar Danish population in which −0.62 and 1.0 represent the worst and best health statuses possible, respectively (Sørensen et al., 2009).
- Cold intolerance was assessed by the Cold Intolerance Symptom Severity (CISS) questionnaire (a scale of 4–100 points, where a higher number indicates worse symptoms) (Irwin et al., 1997). A CISS score of 50 has been determined as the cut-off abnormal value (Carlsson et al., 2010; Stjernbrandt et al., 2021).
- Appearance was assessed by the Michigan Hand Questionnaire (MHQ) aesthetic domain (scale of 0–100, where a higher number indicates better appearance) (Chung et al., 1998).

The patients rated the overall function and appearance in their injured upper extremity on two numeric rating scales (NRS) from 0 to 10 (0 worst, 10 best). We also asked patients to report in free text their most disabling symptom. We classified their answers into one, two or all three of the following categories: pain; functional disability; and an unpleasant appearance. The patients were asked about their working status before injury, return to work and whether they used protheses or gripping aids.

### Statistics

We have presented continuous data for patient characteristics as means with standard deviations (SDs), but PROM data as medians and interquartile ranges (IQRs) as the data for most of these were skewed. By a post hoc power analysis, the sample size would require 35 patients in each group to perceive a 10-point difference in DASH score at a power of 80%, with a standard deviation of 15 points for a two-sided test, at a significance level <0.05. We used previous studies of major upper extremity amputations to estimate variation in DASH scores (Assouline et al., 2017; Mattiassich et al., 2017; Ng et al., 2014; Pet et al., 2016; Resnik et al., 2019; Stanger et al., 2015). Our sample sizes were thus too small for credible statistical testing of differences between the subgroups of patients according to treatment and injury level.

## Results

Among 2250 upper extremity amputations during the 11-year study period, we identified 38 patients with an amputation through or proximal to the carpus. All were invited to participate in the study and 31 responded. Of these, 20 patients had a successful replantation, two had a failed replantation and nine had a primary revision. The patient demographic characteristics are presented in Table 1. Two patients were aged under 18 years at the time of the accident (range 10–79), but all patients were aged above 18 years at the time of assessment (range 19–84). The median number of secondary operations was 1 (IQR: 0–2) after a successful replantation, 3 (IQR: 2–3) after a failed replantation attempt and 0 (IQR: 0–1) after a primary revision (completion) amputation. Two patients had a revascularization procedure and two patients underwent a secondary revision amputation after a failed replantation attempt. Most secondary operations were related to a soft tissue revision, reconstruction or skin grafting, but there were also some operations for fracture nonunion or malunion. One patient had sepsis after replantation. The patients who were treated with a primary revision amputation had no complications. The minimum follow-up time was 21 months with a mean of 8 years (SD 4). After a revision amputation most patients (7 of 11) used a prosthesis and two patients reported the use of a gripping aid after replantation.

**Table 1.** Demographics.

	Replantation/revascularization (*n* = 20)	Revision amputation (*n* = 11)
Age at follow-up (years)	47 (19)	47 (20)
Duration of follow-up (years)	9 (4)	7 (4)
Sex		
Male	14	8
Female	6	2
Dominant hand involved	13	9
Mechanism of injury		
Cut	10	1
Crush	4	3
Avulsion and others	6	7
Amputation type		
Total	12	8
Subtotal	8	3
Occupational accident	3	3
Smoking	6	1
Comorbidities		
Dyslipidaemia	0	2
Diabetes mellitus	2	1
Arterial hypertension	7	2
Amputation groups		
Distal to the elbow joint	16	5
Proximal to the elbow joint	4	6

Data are expressed as *n* or mean (SD).

The PROM data showed that the patients in both groups had considerable disability (Table 2). In total, 14 patients with a successful replantation and eight with a revision amputation had DASH scores over 20 points. Five patients with a successful replantation and three patients with a revision (completion) amputation had a CISS of more than 50 points.

**Table 2.** Treatment outcomes after replantation/revascularization and revision amputation.

	Replantation/revascularization		Revision amputation	
	*n* ^a^	Median (IQR)	*n* ^a^	Median (IQR)
Total	20		11	
DASH	19	30 (21–47)	11	33 (16–52)
EQ-5D-5L index	20	0.7 (0.6–0.9)	11	0.7 (0.7–0.8)
EQ-VAS	20	75 (50–85)	10	73 (70–80)
CISS	14	33 (29–64)	6	52 (26–57)
MHQ aesthetic domain	17	63 (38–81)	10	47 (27–73)
NRS rating of appearance	20	8 (5–10)	10	8 (5–9)
NRS rating of function	20	6 (3–8)	10	1 (0–8)
Distal to the elbow joint	16		5	
DASH	15	30 (21–46)	5	10 (10–22)
EQ-5D-5L index	16	0.8 (0.7–0.9)	5	0.7 (0.7–0.8)
EQ VAS	16	80 (50–85)	5	80 (70–90)
CISS	13	34 (30–67)	3	19 (19–39)
MHQ aesthetics	15	63 (34–84)	5	56 (25–75)
NRS rating of appearance	16	8 (5–10)	5	8 (7–9)
NRS rating of function	16	6 (3–8)	5	8 (0–8)
Proximal to the elbow joint	4		6	
DASH	4	35 (18–46)	6	48 (36–53)
EQ-5D-5L index	4	0.6 (0.5–0.7)	6	0.7 (0.7–0.7)
EQ VAS	4	65 (53–76)	5	70 (70–75)
CISS	1	15 (–)	3	55 (52–57)
MHQ aesthetics	2	72 (67–77)	5	38 (31–69)
NRS rating of appearance	4	9 (7–10)	5	6 (5–8)
NRS rating of function	4	6 (2–9)	5	1 (0–1)

a There are lower numbers for some outcomes as not all patients completed all items in the patient-reported outcome measures, making calculation of their scores impossible.

CISS: Cold Intolerance Symptom Severity (0–100, in which 0 indicates no symptoms); DASH: Disabilities of the Arm, Shoulder, and Hand (0–100, in which 0 indicates no disability); EQ-5D index: the EuroQol EQ-5D-5L index value (0–1, in which 1 indicates the best situation); EQ VAS: the EuroQol EQ-5D-5L health state with visual analogue scale value (0–100, in which 100 indicates the best situation); MHQ aesthetic: Michigan Hand Outcomes Questionnaire aesthetic domain (0–100, in which 100 indicates the best situation); NRS: numerical rating scale (0–10, where 10 indicates the best score).

A total of 17 patients (12 with replantations and five revision amputations) answered the open question about their most disabling symptom. In patients treated with replantation, the most disabling symptoms were related to functional disability (10/12), while pain (2/12) and an unpleasant appearance (1/12) were less common issues. In patients treated with revision amputation, the most disabling symptoms were related to functional disability (4/5), pain (2/5) and an unpleasant appearance (3/5). An analysis of individual responses to DASH questions found no specific problems and indicated a wide range of disability issues in both treatment groups. Data on return to previous work are presented in Table 3.

**Table 3.** The patients’ working status before the amputation and return to work after treatment.

	Replantation/revascularization (*n* = 20)	Revision amputation (*n* = 11)
Working patients (*n* = 17)		
Return to the same work	3	4
Return to different work	5	1
Unable to return to work	3	1
Non-working patients (*n* = 14)		
Retired	6	4
Student	2	1
Unemployed	1	0

## Discussion

We found that after upper limb amputation, most patients had a major disability, cold intolerance, low hand function and appearance ratings, and incapacity for work regardless of the treatment type.

We used PROMs to quantify outcomes that were most important for the patients. Self-rated measures can, however, be influenced by psychological factors, such as pain catastrophizing or coping with disability. Our use of DASH, a broad and generic PROM that is the most widely used in orthopaedic research to quantify upper extremity function, provided the possibility for comparisons with other amputation studies and to different conditions and injuries. Nevertheless, the DASH does not assess all the problems that may be important to the patients. Therefore, we also included validated PROMs for cold intolerance and aesthetics, and NRS ratings of hand function and appearance.

Previous studies have reported mean DASH scores in the range of 25–75 after a major upper extremity amputation injury (Assouline et al., 2017; Mattiassich et al., 2017; Ng et al., 2014; Pet et al., 2016; Resnik et al., 2019; Stanger et al., 2015). Pet et al. (2016) have reported major upper extremity replantation and prosthetic rehabilitation patients to have mean DASH scores of 25 and 40 points, respectively. Our DASH results agree with those, and our further assessment of the symptoms show that there is much variation in the presentation of symptoms. Otto et al. (2015) conducted a systematic review, which concluded that replantation was associated with better outcomes after a major upper extremity amputation, especially in the domains of global satisfaction and psychological well-being.

Chronic phantom limb pain is common, with a prevalence in the range of 7%–49% after upper extremity amputations (Tintle et al., 2010). In our study, the most disabling symptoms that patients rated in open questions were related to functional disability and only approximately one in four patients reported pain to be the most disabling symptom. However, many patients had abnormal CISS scores (>50) and the assessment of the individual DASH questions showed relatively high scores on the pain items. These findings emphasize the importance of pain-related problems after major upper extremity amputations. Pain is connected with psychological coping and self-confidence (Maddison et al., 2022), in accordance with the participants’ EQ-5D scores in our study.

After a revision amputation, most patients used a prosthesis. Continued use can be considered as a measure of perceived benefit from a prosthesis (Smail et al., 2021). Prosthesis users have reported a better quality of life and higher employment rates when compared with non-users, but the quantity of direct functional benefits of an upper extremity prosthesis is unclear (Kerver et al., 2022; Postema et al., 2016; Resnik et al., 2020; Yamamoto et al., 2019).

Most of the working patients could not return to their previous occupations. Other studies have shown a similar reduction in the ability to work (Assouline et al., 2017; Ng et al., 2014; Pet et al., 2016; Ramji et al., 2020). Thus, major upper extremity amputations cause not only individual disability but also a considerable economic cost to society as a result of reduced ability to work.

The major limitation of our study is the relatively small sample size with heterogeneous injuries, which increases random variation and decreases the generalizability of the study results. Because of the small sample size and non-random allocation into the treatment groups, a direct comparison between the two treatment groups was not justified. However, the aim of this study was to describe patient-rated treatment outcomes of proximal amputation patients, not to compare treatment algorithms. We believe the study size to be reasonable based on the low incidence of these injuries, the consecutive inclusion of all patients within a defined geographical region, and the high participation rate. The Finnish population is covered by free, universal healthcare, which decreases selection bias and increases the generalizability of our results.

In summary, our study shows that major upper extremity amputation had a marked effect on health and abilities in daily life, even after a technically successful replantation. The symptoms related to functional disability were rated the most significant, but pain and aesthetic concerns were also considerable.
